# NRF2 attenuation aggravates detrimental consequences of metabolic stress on cultured porcine parthenote embryos

**DOI:** 10.1038/s41598-024-53480-8

**Published:** 2024-02-05

**Authors:** Werner Giehl Glanzner, Leticia Rabello da Silva Sousa, Karina Gutierrez, Mariana Priotto de Macedo, Luke Currin, Felipe Perecin, Vilceu Bordignon

**Affiliations:** 1 Department of Animal Science, McGill University, 21111, Lakeshore Road, Sainte Anne de Bellevue, QC H9X 3V9 Canada; 2 Veterinary Medicine Department, College of Animal Science and Food Engineering, University of São Paulo (USP), Pirassununga, SP Brazil

**Keywords:** Cell biology, Developmental biology

## Abstract

The nuclear factor erythroid 2–related factor 2 (NRF2) is a crucial transcription factor that plays a central role in regulating oxidative stress pathways by binding antioxidant response elements, but its involvement in early embryo development remains largely unexplored. In this study, we demonstrated that NRF2 mRNA is expressed in porcine embryos from day 2 to day 7 of development, showing a decrease in abundance from day 2 to day 3, followed by an increase on day 5 and day 7. Comparable levels of NRF2 mRNA were observed between early-cleaving and more developmental competent embryos and late-cleaving and less developmental competent embryos on day 4 and day 5 of culture. Attenuation of NRF2 mRNA significantly decreased development of parthenote embryos to the blastocyst stage. When NRF2-attenuated embryos were cultured in presence of 3.5 mM or 7 mM glucose, development to the blastocyst stage was dramatically decreased in comparison to the control group (15.9% vs. 27.8% for 3.5 mM glucose, and 5.4% vs. 25.3% for 7 mM glucose). Supplementation of melatonin moderately improved the development of NRF2-attenuated embryos cultured in presence of 0.6 mM glucose. These findings highlight the importance of NRF2 in early embryo development, particularly in embryos cultured under metabolically stressful conditions.

## Introduction

Redox events are natural outcomes of cell metabolism, however, an imbalance in redox state can disrupt normal cellular homeostasis. Oxidative stress and the generation of reactive oxygen species (ROS) occur when molecules capable of oxidizing essential cellular components accumulate^1^. Oxidants can be generated endogenously through oxidase enzymes or the mitochondrial electron transport chain, or they can originate from exogenous sources such as nutrients, drugs, toxic compounds, pollutants, and radiation^2,3^. Early embryonic processes necessitate high energy levels, and embryonic cells are particularly sensitive to oxidants. In porcine embryos, over 90% of ATP is produced through oxidative phosphorylation^4^, which increases ROS production. Porcine oocytes are abundant in lipids^4^, and reducing their lipid content by supplementing L-carnitine in the maturation medium has been shown to enhance embryo development and cryotolerance^5–7^. This suggests early developing embryos utilize lipids as an energy source^8^, potentially overloading mitochondria and oxidative phosphorylation process, leading to increased ROS production. High levels of oxidative stress and ROS can impact cellular signaling pathways involved in cell proliferation, differentiation, apoptosis, and DNA repair^9^. It is well-established that early-stage embryos do not efficiently metabolize glucose^10^, and continuous glucose supplementation throughout in vitro culture of porcine embryos impairs normal embryo development^11,12^, exacerbating oxidative stress and endoplasmic reticulum stress. The effects of metabolic disorders, such as diabetes, alongside an increase in tissue-specific oxidative stress, have a significant impact not only on embryos but also on adult metabolic and reproductive functions, such as ovary/oocyte aging and polycystic ovary syndrome (PCOS)^13–15^. These disorders appear to benefit from the use of antioxidants^16^, especially melatonin^17^.

Melatonin is a widely used antioxidant for culturing embryos in vitro^18–22^. Its receptor is expressed in the female reproductive tract, conceptus, and endometrium^23,24^. Melatonin has been suggested to mitigate fertility decline and reduce ovarian aging^25^. It has also been shown to improve the developmental competence of porcine oocytes and embryos^18,19,21,26,27^. Additionally, melatonin has been found to modulate histone acetylation levels^18^, and regulate lipid metabolism by influencing the expression of key genes involved in lipid metabolism, ATP content, and lipid droplet size^26^. There is evidence indicating that melatonin also plays a role in glucose metabolism^20^. Its supplementation during in vitro culture of mouse embryos has been shown to regulate the insulin signaling pathway by inhibiting RhoA and increasing hepatic Fbxl7 expression, both of which are associated with glucose metabolism. Many of the effects of melatonin are attributed to its ability to alleviate oxidative stress and are proposed to be mediated through NRF2, either directly or indirectly^28–31^.

NRF2 is a master regulator of oxidative balance and plays various cellular roles in regulating redox homeostasis, mitochondrial function, oxidative phosphorylation, ATP production, and fatty acid oxidation^32^. NRF2 has been shown to regulate oxidative stress in granulosa cells under stressful conditions^33^, and this function may involve interactions with miRNAs^34^. Furthermore, NRF2 induces peroxisomal activities in porcine oocytes and embryos^31,35^. The NRF2 signaling pathway involves numerous targets and regulates over 600 genes^36^, including more than 200 cytoprotective agents like heme-oxygenase 1 (HO1) and SIRT1. NRF2 expression appears to be correlated with the levels of two other antioxidant genes, Superoxide Dismutase 1 and 2 (SOD1 and SOD2)^29,30,37,38^. SOD1 has been extensively studied in neurological disorders such as Amyotrophic Lateral Sclerosis (ALS) and Parkinson’s disease, as well as cancer^39^. Similarly, SOD2 has been associated with cancer, metastasis^40^ and mitochondrial dysfunction leading to neurodegeneration^41^. However, their roles during early embryo development remain unclear. The primary negative regulator of NRF2 is KEAP1, which facilitates the degradation of NRF2^36,42,43^. Various molecules have been shown to stimulate NRF2 in reproductive tissues and cells, thereby reducing oxidative stress, such as quercetin^37^, N-acetylcysteine^44^, α-ketoglutarate^45^, sulforaphane^43,46^ and melatonin^28–30^.

Given the pivotal role of NRF2 in maintaining oxidative balance, this study aimed to investigate its importance in porcine embryo development. First, the levels of NRF2 mRNA were determined in porcine embryos at different developmental stages and compared between embryos exhibiting high and low developmental competence. Second, NRF2 mRNA expression was attenuated, and the impact on embryo development was assessed under standard and stressful culture conditions. Lastly, the association between NRF2 and melatonin was evaluated by culturing NRF2-attenuated embryos in the presence of melatonin, under both control and stressful culture conditions.

## Results

### mRNA for antioxidant regulators is differently expressed during early embryo development

**Figure 1:**
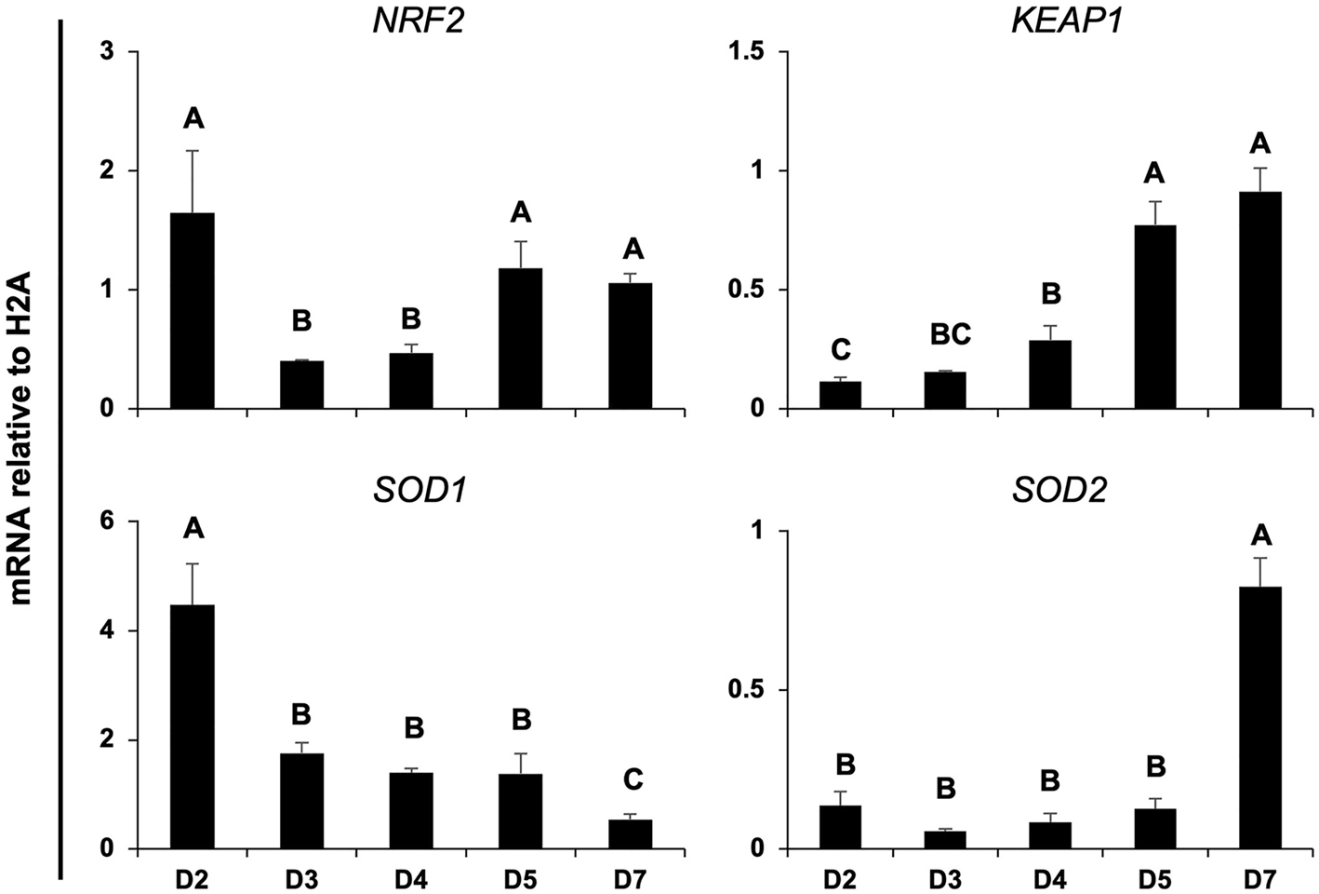
mRNA expression levels of NRF2, KEAP1, SOD1 and SOD2 in porcine IVF embryos at different developmental stages from day 2 to day 7. Statistical differences (*p* < 0.05) in the relative mRNA abundance between days for each gene are indicated by different capital letters above bars. Samples used for RNA extraction were obtained from three independent replicates, with each sample containing 10–15 embryos. Results are presented as means ± SEM.

**Figure 2:**
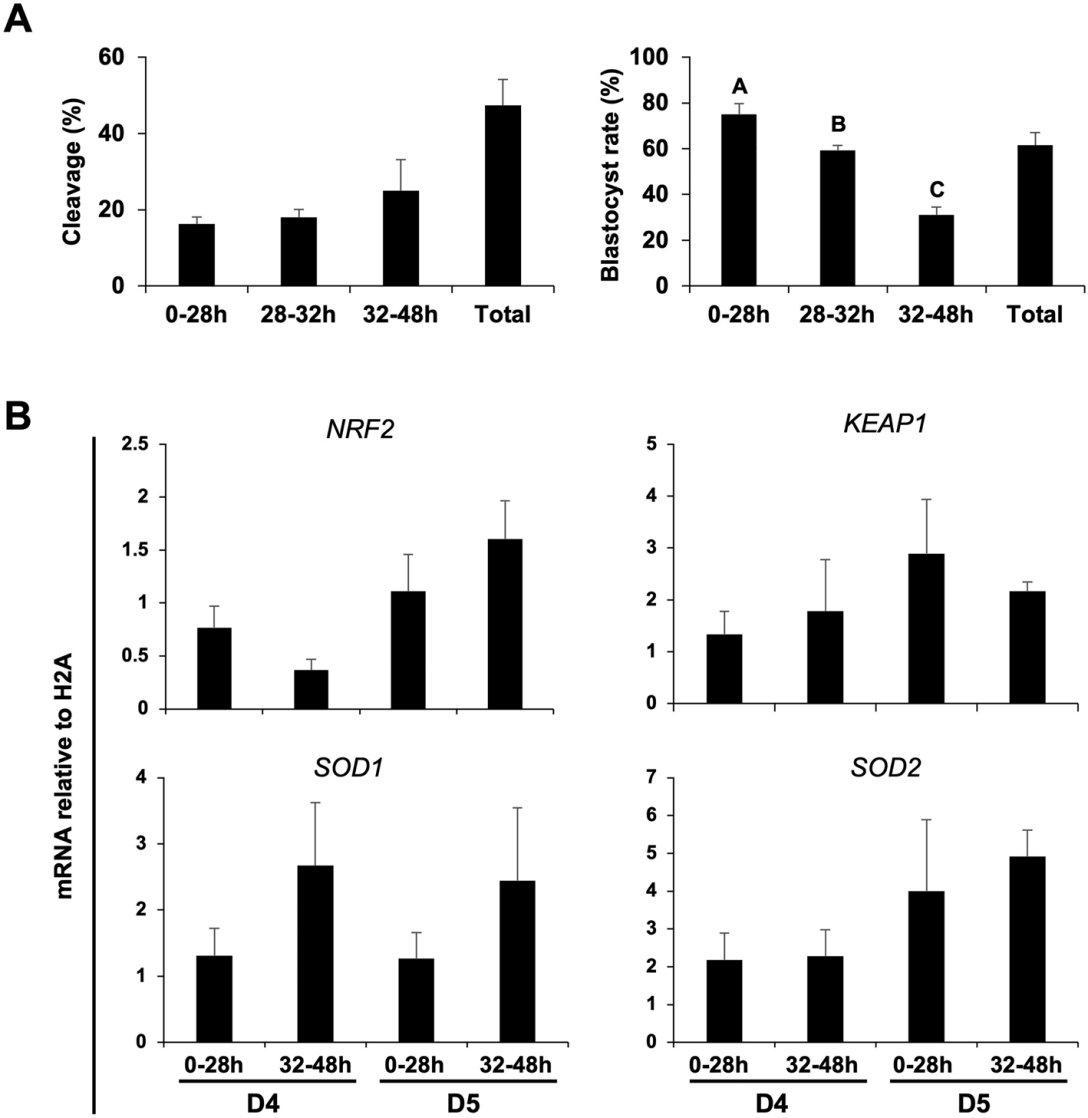
Developmental rates and mRNA expression of oxidative stress regulators in partheno-genetically activated embryos with different cleavage kinetics. (**A**) Cleavage rates were evaluated during the time intervals of 0–28 h, 28–32 h and 32–48 h post-activation, and blastocyst rates were assessed on day 7 of development. (**B**) Transcript levels of NRF2, KEAP1, SOD1, and SOD2 in early- (before 28 h) and late-cleaving (32-48 h) embryos on day 4 and day 5 of development. Data were obtained from three independent replicates. For mRNA level assessment, each replicate consisted of 10–15 embryos per sample. Statistical differences (*p* < 0.05) in blastocyst rates between cleavage groups are indicated by different capital letters above bars. Results are presented as means ± SEM.

To evaluate if the NRF2 system is present during porcine early embryo, pools of embryos produced by IVF were collected on day 2 (D2), day 3 (D3), day 4 (D4), day 5 (D5) or day 7 (D7) of development to assess mRNA of NRF2, its regulator KEAP1, SOD1 and SOD2 levels in porcine embryos (Fig. 1). NRF2 mRNA levels decreased on D3 and D4 compared to D2, then increased on D5 and remained high on D7 embryos (Fig. 1). KEAP1 mRNA levels increased steadily between D2 and D7 of development (Fig. 1). A contrary pattern was observed for SOD1 mRNA, which decreased between D2 and D7 of development (Fig. 1), and SOD2 mRNA, which remained low between D2 and D5 embryos but was dramatically increased on D7 embryos (Fig. 1). The observed mRNA expression pattern of these regulators suggests their relative contribution as antioxidant regulators may vary according to the embryo developmental stage. Next, we assessed if mRNA expression for these antioxidant regulators was differently expressed in porcine embryos having high and low developmental competence, as determined by the time from activation to their first cleavage^47–49^. Early-cleaving embryos (< 28 h post-activation) had superior development than intermediate (28–32 h post-activation), and late cleaving (32–48 h post-activation) (Fig. 2A). However, mRNA levels of NRF2, KEAP1, SOD1 and SOD2 were not differently expressed between early and late cleaving embryos on day 4 (D4) or day 5 (D5) of development (Fig. 2B). These timepoints of development were selected because results from the first experiment revealed that transcripts levels for NRF2 and KEAP1 were increased on D5 embryos compared to D4 embryos.

### NRF2 is important for embryo development to the blastocyst stage

**Figure 3:**
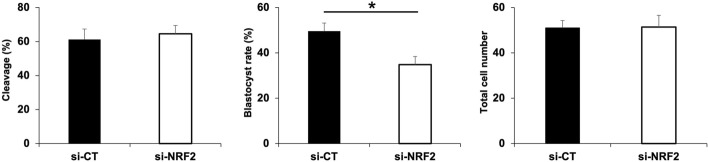
Effect of attenuating NRF2 mRNA on embryo development. Cleavage rates, blastocyst rates, and total cell numbers in control embryos (si-CT; scramble sequences) and RNF2-attenuated (si-NRF2) embryos produced by parthenogenetic activation. Data are from three independent replicates. The asterisk indicates a statistically significant difference (*p* < 0.05) in blastocyst rates between the two treatments. Results are presented as means ± SEM.

**Figure 4:**
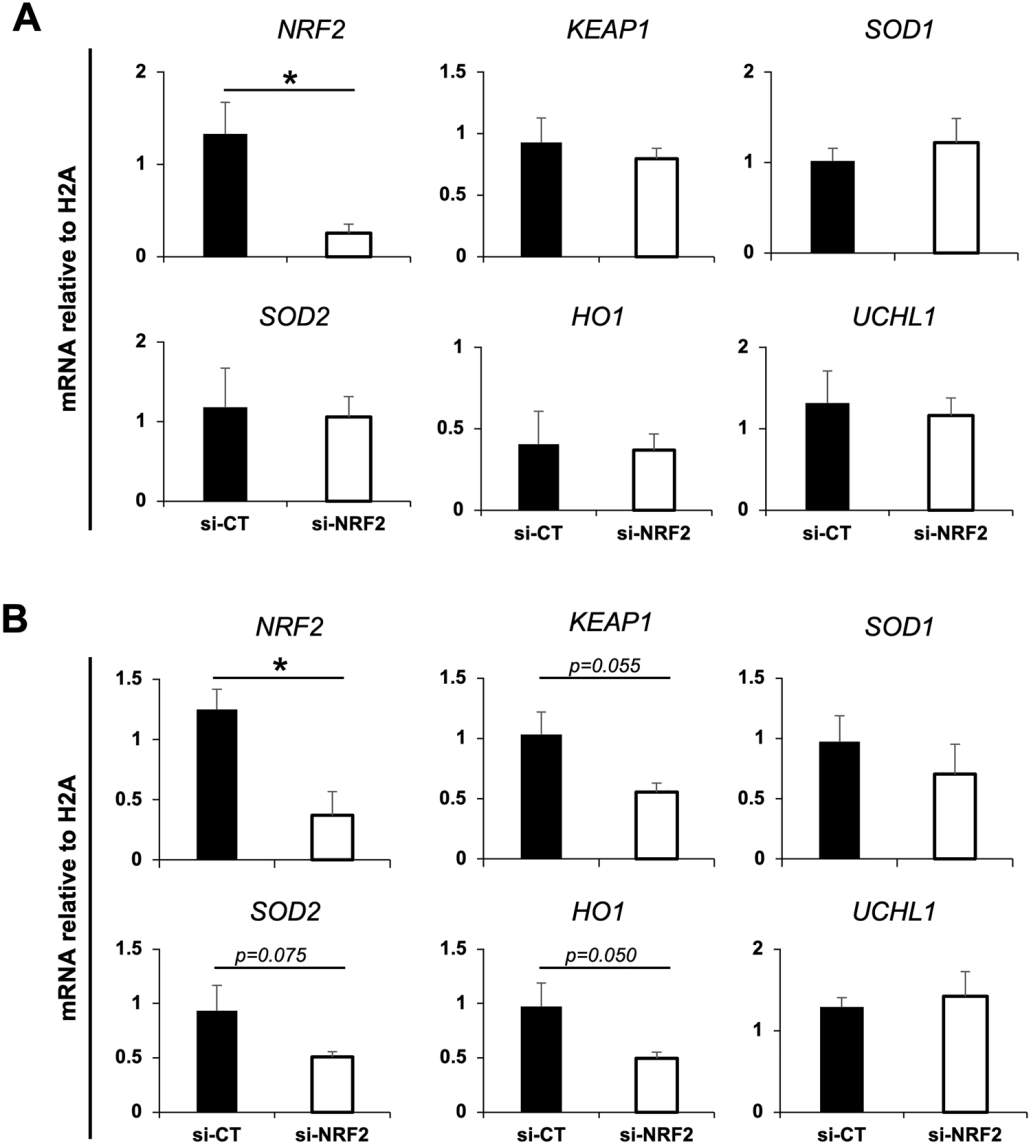
Transcript levels of genes involved in oxidative stress regulation. Relative mRNA abundance of NRF2, KEAP1, SOD1, SOD2, HO1 and UCHL1 on day 3 (D3; Panel **A**), and day 5 (D5; Panel **B**) of development in control embryos (si-CT) and RNF2-attenuated (si-NRF2) embryos produced by parthenogenetic activation. Samples used for RNA extraction were obtained from three independent replicates, with each sample containing 10–15 embryos. Asterisks indicate statistically significant differences (*p* < 0.05) in the relative mRNA abundance between the two treatments. Results are presented as means ± SEM.

Considering its known role as a master regulator of antioxidant response, we have focused on the characterization of NRF2 importance for early development of porcine embryos. To avoid potential impacts of abnormal fertilization often associated with IVF in the porcine species, all experiments involving NRF2 attenuation utilized parthenogenetic activated (PA) embryos. We found that NRF2 mRNA attenuation, via microinjection of DsiRNAs (si-NRF2) into metaphase II oocytes following by their activation, did not decrease cleavage rates but reduced blastocyst rates (Fig. 3) compared to controls injected with DsiRNAs scramble sequences (si-CT). The total number of cells in embryos that reached blastocyst stage were similar between si-NRF2 and si-CT groups (Fig. 3). Quantification of mRNA levels by qPCR confirmed NRF2 attenuation by 80% on D3 embryos (Fig. 4A) and 70% on D5 embryos (Fig. 4B), compared to controls. While NRF2 attenuation did not affect transcript levels of KEAP1, SOD1, SOD2, HO1 and UCHL1 on D3 embryos (Fig. 4A), mRNA levels of KEAP1, SOD2 and HO1 tended to decrease in NRF2-attenuated embryos on D5 of development (Fig. 4B) compared to controls. These results indicated that NRF2 is important for early embryo development but is not required for development to the blastocyst stage of porcine embryos under normal culture conditions.

### NRF2 is required for the embryo coping response to detrimental culture conditions

**Figure 5:**
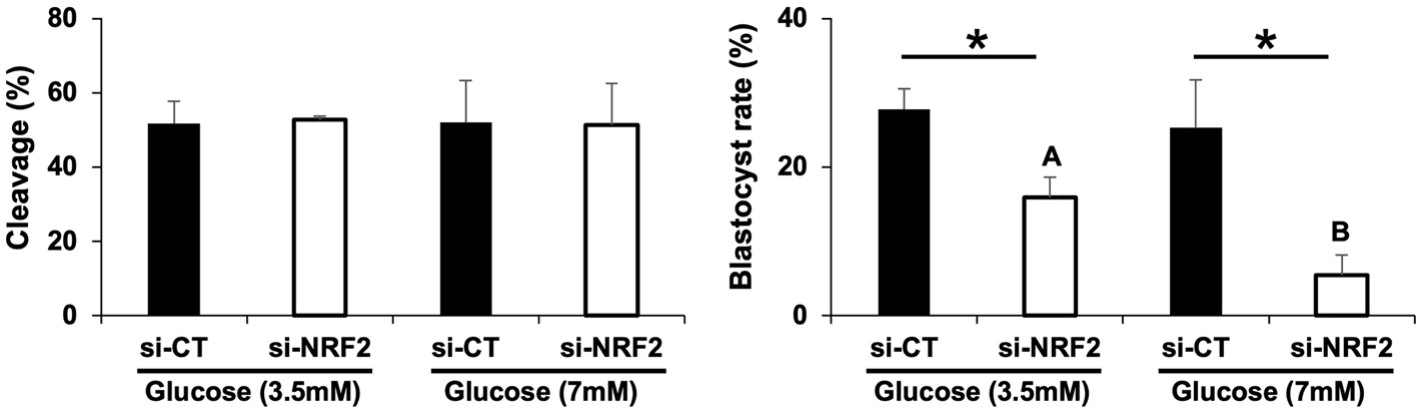
Development of embryos cultured with excess glucose. Cleavage rates and blastocyst rates of control PA embryos (si-CT) and RNF2-attenuated PA embryos (si-NRF2) embryos treated with either 3.5 or 7 mM of glucose. Data are from three independent replicates. Asterisks indicate statistically significant differences (*p* < 0.05) in blastocyst rates between the two treatments. Different capital letters above bars indicate statistically significant differences (*p* < 0.05) in blastocyst rates between the two glucose concentrations in the RNF2-attenuated embryos. Results are presented as means ± SEM.

**Figure 6:**
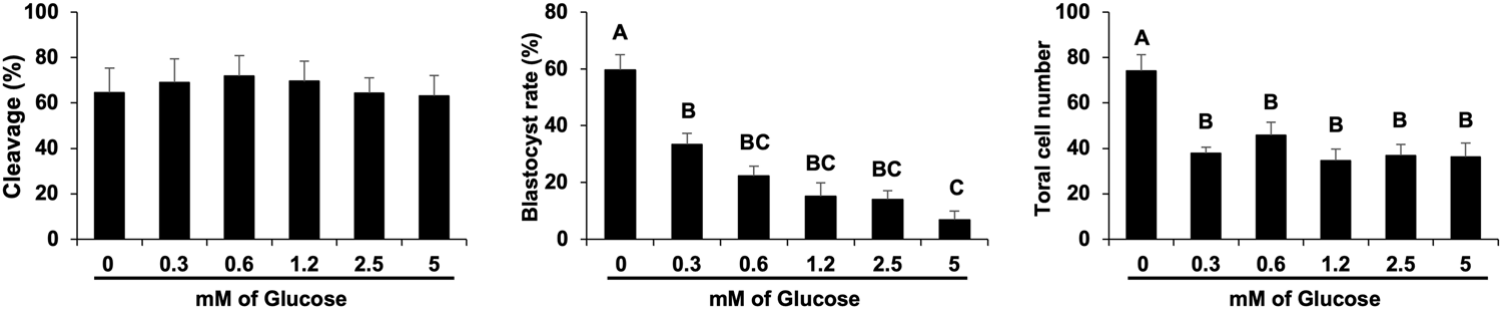
Development of PA embryos cultured with different concentrations of glucose. Cleavage rates, blastocyst rates, and total cell numbers of embryos treated with various concentrations of glucose. Data are from three independent replicates. Different capital letters above bars denote statistically significant differences (*p* < 0.05) in blastocyst rates or total cell numbers between the treatments. Results are presented as means ± SEM.

To investigate if NRF2 would regulate embryo coping responses if cultured under stressful conditions, si-CT and si-NRF2 injected embryos were cultured in presence of excess glucose (3.5 mM or 7 mM). Cleavage rates of NRF2-attenuated embryos were not affected by culture with excess glucose (Fig. 5). However, blastocyst rates of NRF2-attenuated were significantly decreased by both glucose concentrations, with a more prominent impact of the higher concentration (Fig. 5). This observation indicates that NRF2 plays an important role in sustaining early embryo development if embryos are cultured under a metabolic stressful environment. Proving that both glucose concentrations tested were highly detrimental for embryo development, a dose response experiment was performed and revealed a steadily decrease in blastocyst rates as the glucose concentration in culture was increased from 0 to 5 mM (Fig. 6). Even the lower dose tested (0.3 mM) significantly decreased blastocyst rates and quality, as indicated by the lower number of cells in embryos that reached the blastocyst stage (Fig. 6). Both blastocyst rates and total number of cells per blastocyst were not statistically different between embryos that were treated with 0.6 mM or higher concentrations of glucose (Fig. 6). Based on these results, we opted to use the concentration of 0.6 mM glucose in the subsequent experiments.

### Melatonin partially rescues NRF2 attenuation impact in embryos cultured under detrimental culture conditions

**Figure 7:**
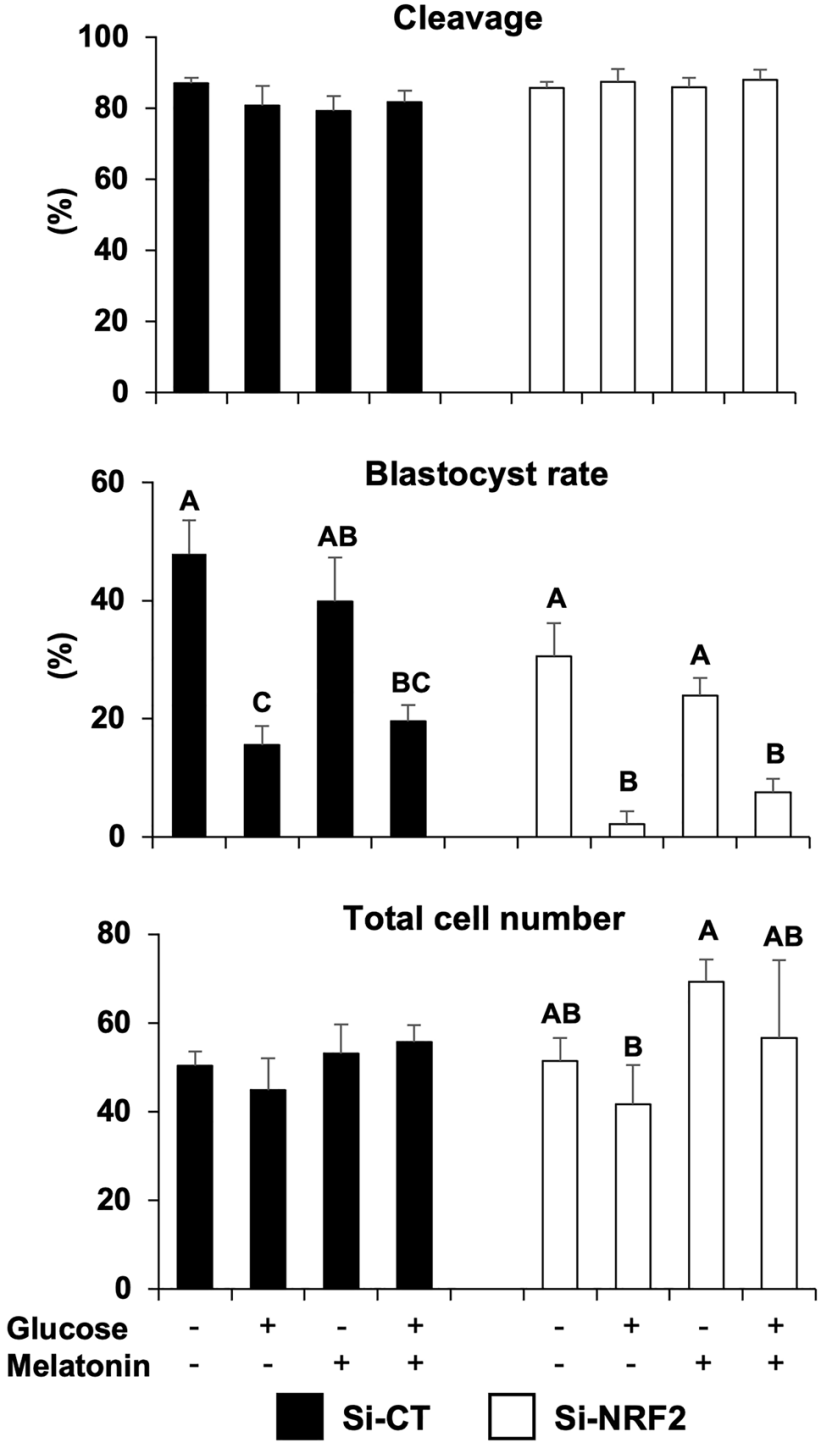
Development of PA embryos cultured with glucose and melatonin. Cleavage rates, blastocyst rates, and total cell numbers of control embryos (si-CT) and RNF2-attenuated (si-NRF2) embryos cultured with either glucose (0.6 mM), melatonin (100 nM), or a combination of both (glucose + melatonin). Data are from three independent replicates. Different capital letters above bars indicate statistically significant differences (*p* < 0.05) in blastocyst rates or total cell numbers between the treatments within the si-CT and si-NRF2 groups. Results are presented as means ± SEM.

**Figure 8:**
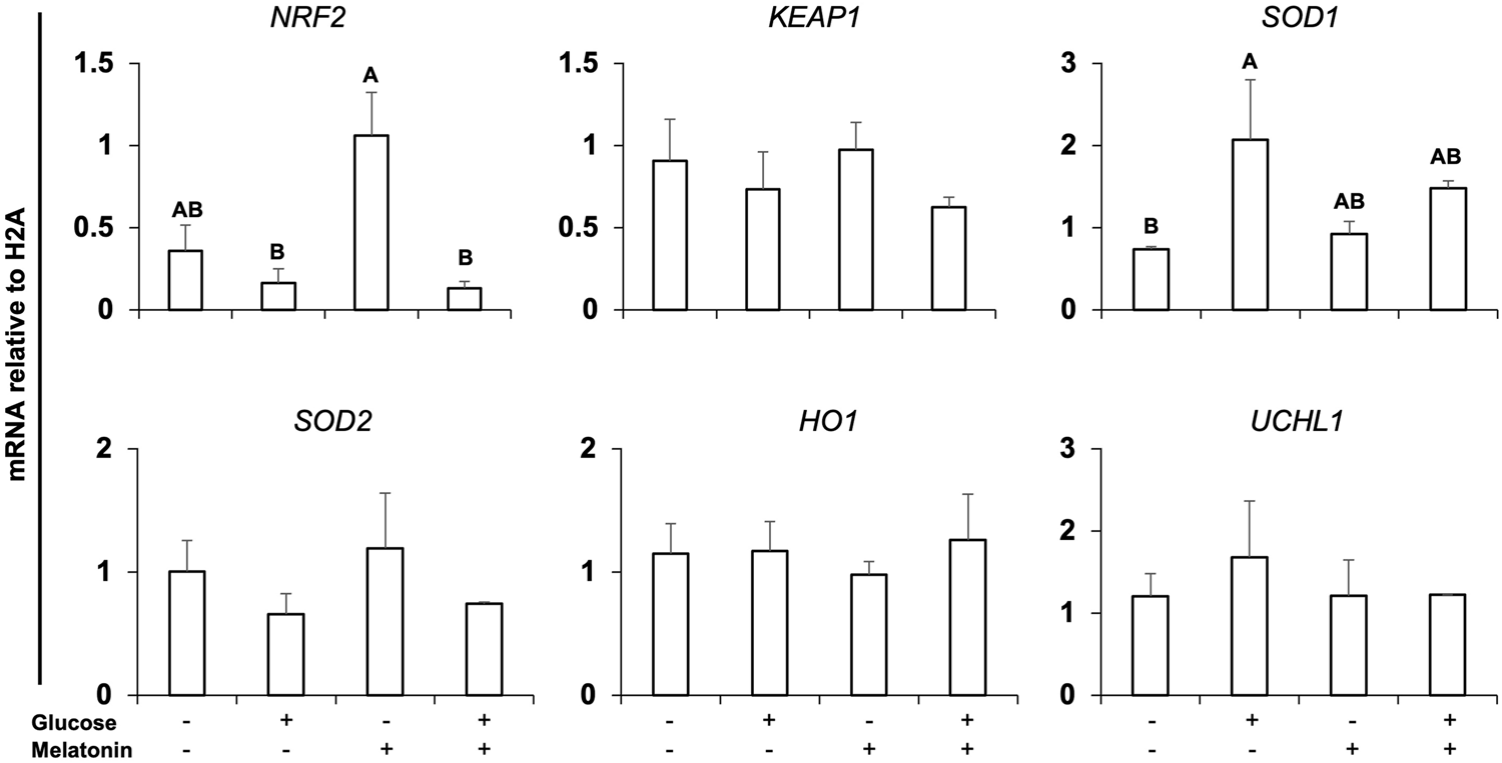
Transcript levels of genes involved in oxidative stress regulation in RNF2-attenuated PA embryos. Relative mRNA abundance of NRF2, KEAP1, SOD1, SOD2, HO1 and UCHL1 on day 5 of development in RNF2-attenuated embryos cultured with either glucose (0.6 mM), melatonin (100 nM), or a combination of both (glucose + melatonin). Samples used for RNA extraction were obtained from three independent replicates, with each sample containing 10–15 embryos. Different capital letters above bars indicate statistically significant differences (*p* < 0.05) in the relative mRNA abundance between the treatments. Results are presented as means ± SEM.

We tested if supplementation of melatonin during embryo culture could alleviate the negative impact of NRF2 attenuation once previous studies suggested that melatonin action is dependent on NRF2 signaling^28,29^. Embryos were injected with either si-CT or si-NRF2 and then cultured in presence of either glucose (0.6 mM), melatonin (100 nM), or both (0.6 mM glucose + 100 nM melatonin). Cleavage rates were not affected by treatment (Fig. 7). Blastocyst rates in melatonin-treated embryos were similar to control group, while glucose-treated embryos had lower development in both si-CT and si-NRF2 injected groups (Fig. 7). Melatonin treatment resulted in a numerical but not statistically significant improvement of embryo development (Fig. 7). Melatonin treatment also showed a numerical but not statistically different increase in the total cell number per blastocyst in NRF2-attenuated embryos that were cultured in either presence or absence of glucose (Fig. 7). Finally, the effect of melatonin and glucose on the mRNA levels of NRF2, KEAP1, SOD1, SOD2, HO1 and UCHL1 were assessed in NRF2-attenuated embryos on D5 of development. Treatment with melatonin in absence of glucose resulted in an upregulation of NRF2 mRNA levels compared to groups treated with glucose, regardless of melatonin presence (Fig. 8). In addition, glucose treatment upregulated SOD1 mRNA levels in comparison with the control group, and this effect was mitigated by melatonin supplementation in the culture medium (Fig. 8).

## Discussion

Normal embryo development relies on proper cellular homeostasis, but oxidative stress significantly impacts this process. Various factors may contribute to cellular and oxidative stress during embryo culture including oxygen tension^50^, glucose concentration^12^, light, temperature, culture media supplements and culture equipment^51^. Increased stress levels during embryo culture can lead to detrimental effects such as increased genomic instability^52^ and endoplasmic reticulum (ER) stress^12,52^, which can activate the unfolded protein response (UPR) and trigger cell death^53^. Therefore, gaining a deeper understanding of cellular responses to conditions that elevate oxidative stress is crucial for developing enhanced culture environments and generating healthy embryos and offspring. Previous studies have suggested that NRF2 serves as a critical mediator of the oxidative stress response in embryos^29,35,54^. However, the importance and role of NRF2 in porcine embryo development under a metabolically stressful environment has not been characterized through its specific inhibition with RNAi.

Initially, we confirmed the presence of mRNA for NRF2 and other antioxidant regulators, KEAP1, SOD1 and SOD2, in porcine embryos from cleavage stage (D2) to the blastocyst stage (D7), indicating the significance of redox reactions and oxidative homeostasis in early embryo development. Utilizing a well-established model of embryo developmental competence, based on the time required for the first cell cleavage^12,47–49,55^, we observed that transcript levels of NRF2, KEAP1, SOD1 and SOD2 were comparable between early- and late-cleaving embryos on days 4 and 5 of development. While previous studies have identified differences between early- and late-cleaving embryos in various aspects, including oxidative stress^55,56^, ER stress^49^, DNA damage^48^, and epigenetic regulators^47^, our findings suggest that the expression of crucial regulators of oxidative stress is not significantly altered in embryos with lower developmental capacity. However, it is worth highlighting that our experiments were conducted under standard culture conditions, thus it remains unknown whether early-cleaving embryos are better equipped to respond to oxidative challenges than late-cleaving embryos, warranting further investigation.

Next, we found that the attenuation of NRF2 mRNA decreased blastocyst rates in embryos cultured under non-stressful conditions but did not affect total cell number in embryos that reached the blastocyst stage. This observation not only confirmed the crucial role of NRF2 in early embryo development but also indicated its contribution to blastocyst development is limited to a small proportion of embryos, while most embryos having the potential to form a blastocyst can reach that stage independently of NRF2. It is possible that embryos unable to reach the blastocyst stage in absence of NRF2 are either more vulnerable to oxidative stress or are less equipped to cope with oxidative challenges occurring in the culture milieu. Therefore, we further explored the importance of NRF2 in the development of embryos cultured under a metabolically stressful environment induced by excess glucose. Interestingly, the detrimental impact of culture with excess glucose on embryo development was significantly exacerbated in NRF2-attenuated embryos compared to control embryos. This finding indicates that NRF2 plays a crucial role in regulating the embryo's response to adverse culture conditions. Previous studies involving bovine granulosa cells and embryos have also suggested that NRF2 function is primarily required under stressful conditions^33,37^. Moreover, various molecules with protective roles in cultured cells, including retinal epithelial cells^57^ and podocytes^58^, appears to exert their effects through the activation of NRF2, although the underlying molecular pathways have yet to be fully characterized.

Our finding in NRF2-attenuated embryos revealed a tendency for downregulation of KEAP1, HO1 and SOD2 mRNA levels in day 5 embryos, suggesting a potential involvement in mediating NRF2 functions. The downregulation of KEAP1 mRNA was expected, given its established role as a negative regulator of NRF2^36,42,43^. Likewise, the decreased expression of HO1 mRNA can be considered an anticipated consequence of NRF2 attenuation, given the significance of the NRF2/HO1 pathway in orchestrating an oxidative response^36^, and mitigating oxidative stress induced by high glucose levels in type 2 diabetes^59,60^. Similarly, the downregulation of SOD2 in NRF2-attenuated embryos may be linked to the importance of the NRF2/SOD2 pathway in alleviating endoplasmic reticulum stress induced by heat^61^, as well as oxidative stress induced by excess glucose^38^ in somatic cells. These findings, along with other recent studies^62^, suggest that NRF2 plays a role in regulating both oxidative and ER stress responses in embryos, but further studies are necessary to confirm this hypothesis.

Lastly, we observed that supplementation of the culture medium with the antioxidant melatonin was unable to rescue development of NRF2-attenuated embryos cultured in excess glucose. It is well established that antioxidant supplementation improves porcine embryo development^22,50^. Melatonin was chosen as the antioxidant for this study due to its known effects in ameliorating oocyte maturation and embryo development^18,21,27,30^, mitigating the detrimental consequences of excess glucose on embryo development, improving glucose metabolism in adult mice by upregulating hepatic FBXL7 expression^20^, and its proposed role in alleviating oxidative stress through NRF2^28–30^. However, based on the concentration used in our study (100 nM), we were unable to observe a significant impact of melatonin on embryo development and embryo quality, or a clear link between melatonin and NRF2. Nevertheless, a trend towards increased embryo development and embryo cell number was observed in response to melatonin supplementation in embryos cultured with excess glucose, both in control and NRF2-attenuated embryos. These results are somewhat contradictory, as previous studies have reported beneficial effects of melatonin through the NRF2 pathway in diabetic mice and/or high glucose-induced cells in reducing oxidative stress^63–65^. Furthermore, melatonin supplementation led to a decrease in the significant upregulation of SOD1 mRNA induced by culture in excess glucose in NRF2-attenuated embryos. It is possible that a higher concentration of melatonin may be required to fully counteract the detrimental impact of excess glucose on early embryo development, prompting the need for further investigation.

In conclusion, the findings of this study highlight the critical role of NRF2 in the pig embryo's ability to mount a protective response against adverse culture conditions induced by excess glucose. Moreover, the study suggests that the antioxidant effects of melatonin in early developing embryos may not solely rely on NRF2. These results shed light on the involvement of NRF2 and melatonin in mitigating the detrimental effects of metabolic stress. Further investigations are warranted to unravel the complex interplay between NRF2, melatonin, and other potential pathways that contribute to the antioxidant and protective functions in early embryos.

## Methods

Unless stated otherwise, all chemicals were purchased from Millipore Sigma (Sig-ma-Aldrich; Oakville, ON, Canada).

### Oocyte collection and in vitro maturation (IVM)

The ovaries of prepubertal gilts were obtained from a local slaughterhouse (CBCO Alliance, Les Cèdres, QC, Canada) and transported to the laboratory at a temperature of 35 °C in sterile saline solution containing penicillin (100 UI/mL) and streptomycin (10 mg/mL). Cumulus-oocyte complexes (COCs) were retrieved from 3 to 6 mm follicles using a 10 mL syringe and a 20-gauge needle. Only COCs with a minimum of three layers of cumulus cells and homogeneous granulated cytoplasm were selected for IVM. Groups of 30 COCs were subjected to IVM at 38.5 °C in a controlled environment of 5% CO2 and 95% air for a duration of 22 h, using 90 µl of maturation medium. The composition of the maturation medium was as follows: TCM-199 (Life technologies, Burlington, ON, Canada), supplemented with 20% of porcine follicular fluid, 1 mM dibutyryl cyclic adenosine monophosphate (dbcAMP), 0.1 mg/mL cysteine, 10 ng/mL epidermal growth factor (EGF; Life technologies), 0.91 mM sodium pyruvate, 3.05 mM D-glucose, 5 IU/mL hCG (Intervet Canada Corp, Kirkland, QC, Canada), 10 µg/mL FSH (Vetoquinol, Lavaltrie, QC, Canada), and 20 µg/mL gentamicin. Following the initial maturation period, the COCs were transferred to the same IVM medium, but in absence of hCG, FSH and dbcAMP, for an additional 20 to 22 h under the same environmental conditions.

### In vitro embryo production

In vitro fertilization (IVF) was conducted following the protocol outlined by Glanzner et al.^66^. Cumulus cells surrounding matured oocytes were removed by subjecting them to vortexing in TCM-199 HEPES-buffered medium (Life Technologies) supplemented with 0.1% hyaluronidase. Denuded oocytes were subsequently washed in modified Tris-Buffered Medium (mTBM)^67^, containing 2 mM caffeine and 0.2% bovine serum albumin (BSA, fatty acid free), and then fertilized for 5 h in groups of 60–80, in four-well plates with 500 µl medium, using a concentration of 2 × 10^5^ sperm/mL. Parthenogenetic activation (PA) was carried out following the methodology of De Macedo et al.^68^. In brief, matured and previously denuded oocytes were exposed to 15 µM ionomycin for a duration of 5 min in TCM-199 supplemented with 0.2% of BSA. After ionomycin exposure, oocytes were washed in TCM199 (0.2% BSA) and incubated in 200 µM TPEN for 15 min. They were then cultured for 4 h in the presence of 7.5 µg/mL cytochalasin B, diluted in PZM-3 supplemented with 0.3% BSA. Presumptive zygotes derived by either IVF or PA were cultured in PZM-3 medium supplemented with 0.3% BSA in a controlled environment of 5% CO2 and 95% air at a temperature of 38.5 °C. In both the IVF and PA procedures, the medium was supplemented with 10% fetal bovine serum (FBS) on day 5 of culture period. Cleavage rates were evaluated on day 2, and blastocyst rates were determined on day 7 of the embryo culture.

### NRF2 attenuation

Dicer-substrate interfering RNAs (DsiRNAs) were designed using the Custom DsiRNA Design Tool and synthetized by Integrated DNA Technologies (Windsor, ON, CA). To ascertain specificity, the Basic Local Alignment Search Tool (BLAST) provided by National Center for Biotechnology Information (Bethesda, MD, USA) was used. In vitro matured (MII) oocytes were microinjected prior to PA using a FemtoJet 4i system (Eppendorf, Hamburg, Germany). The injection involved the delivery of 10 pL of a 25 µM solution containing both sense and antisense DsiRNAs, which were strategically designed to target two distinct sequences in the mRNA of NRF2 (si-NRF2), while control scrambled sequences (si-CT) (Table S1) were employed as a comparative baseline. The microinjections were carried out in TCM-199 HEPES-buffered medium supplemented with 20 µg/mL gentamicin and 0.2% BSA. The effectiveness of knockdown efficiency was confirmed by quantifying the relative mRNA abundance of NRF2 through real-time quantitative PCR (qPCR) on day 3 of embryo development.

### RNA extraction and reverse transcription quantitative PCR (RT-qPCR)

Total RNA was extracted from pools of 10–15 embryos at either D3 or D5 of development. Complementary DNA was synthetized following the methodology described in a previous publication^66^. For the RT qPCR reactions, a CFX 384 real-time PCR system (BioRad, Hercules, CA, USA) was used. The advanced qPCR mastermix (Wisent Bioproducts, St-Bruno, QC, CA) was utilized for all reactions. Primer sequences (Table S2) were designed based on porcine sequences available in GenBank and were synthesized by IDT. Each sample was analyzed in duplicate, and the relative mRNA abundance was normalized to the mean levels of the internal control gene H2A, using the delta CT method. The PCR efficiency for each gene was calculated using a standard curve dilution approach. All reactions exhibited efficiencies ranging between 90 and 110%, r-square values equal or grander than 0.98, and slope values within the range of − 3.6 to − 3.1. To ensure specificity of the amplified products, dissociation curve analyses were conducted.

### DNA staining and cell counting analysis

Developing embryos were fixed in a 4% paraformaldehyde solution for 15 min, and were then permeabilized by treatment with 1% Triton X-100 solution in PBS for 1 h at 37 °C. Afterward, the samples were transferred into a 10 µg/mL 4,6-Diamidino-2-Phenylindole, Dilactate (DAPI) solution for 20 min and then rinsed in permeabilization solution for 10 min before the embryos were mounted onto slides using Mowiol as the mounting solution. The slides were examined using a Nikon eclipse 80i microscope (Nikon). The imaging process involved capturing pictures at a magnification of 200 × using a Retiga 2000R monochrome digital camera (QImaging, Surrey, BC, Canada). To quantify the total number of cells per embryo (number of nuclei) the ImageJ software was utilized.

### Glucose and melatonin treatments

Glucose was diluted into a stock solution (100 mM) directly into PZM3 and serial dilutions were carried out in PZM3 medium to achieve a range of concentrations spanning from 0.3 mM to 7 mM, according to the experiment. Melatonin was initially diluted into a 1000 × stock solution (100 µM) using DMSO as the solvent. The concentration of 100 nM used in the experiments was reached by diluting the stock solution directly into PZM3 medium.

### Statistical analysis

The collected data were analyzed using the JMP software from SAS Institute Inc. (Cary, NC, USA). Variations in mRNA levels, cleavage rates, blastocyst rates, and total embryo cell numbers were assessed by using the Student’s t-test for experiments involving 2 groups. The effect of the group on the dependent variables was assessed using ANOVA followed by the Tukey-HSD pairwise comparisons of least squares corrected means. Data were tested for normal distribution using the Shapiro–Wilk test. Results are presented as means ± SEM, and *p* < 0.05 was considered statistically significant. A minimum of 3 replicates were performed in all experiments.

### Supplementary Information

Supplementary Tables.

## Data Availability

All data generated or analysed during this study are included in this published article (and its Supplementary Information files).
