# Supplementary material for: NRF2 attenuation aggravates detrimental consequences of metabolic stress on cultured porcine parthenote embryos
Source: Sci Rep. 2024 Feb 5;14:2973. doi: 10.1038/s41598-024-53480-8 (PMC10844622; doi:10.1038/s41598-024-53480-8)
Supplement: Supplementary file 1 — Supplementary Tables. [file 41598_2024_53480_MOESM1_ESM.docx]

Supplementary materials:

**Table S1.** DsiRNAs used for knockdown experiments.

| **Target** | **Sense** | **Antisense** |
| --- | --- | --- |
| *NRF2#1* | GAGAAGUAUUUGACUUCAGUCAACG | CGUUGACUGAAGUCAAAUACUUCUCGA |
| *NRF2#2* | CACUUCCAAAGCAAAAAACAACCTA | UAGGUUGUUUUUUGCUUUGGAAGUGUU |
| *Negative control* | CGUUAAUCGCGUAUAAUACGCGUA | AUACGCGUAUUAUACGCGAUUAACGA |

Design ID based on IDT design tool for targeting *Nrf2*: #1 CD.Ri.131478.13.1, #2 CD.Ri.131480.13.1.

**Table S2.** List of primers and accession number of analyzed genes.

| **Gene** | **Forward Primer** | **Reverse primer** | **Accession Number or reference** |
| --- | --- | --- | --- |
| *H2A* | GGTGCTGGAGTATCTGACCG | GTTGAGCTCTTCGTCGTTGC | [37] |
| *NRF2* | AGCCCAGTCTTCATTGCTCC | CGTGCTAGTCTCAGCAAGGT | [12] |
| *KEAP1* | ACGTGGAGACAGAAACGTGG | GTGTCCGTGTCTGGGTCATA | [12] |
| *SOD1* | AAGGCCGTGTGTGTGCTGAA | GATCACCTTCAGCCAGTCCTTT | [12] |
| *SOD2* | GGCCTACGTGAACAACCTGA | TGATTGATGTGGCCTCCACC | [12] |
| *HO1* | TACCGCTCCCGAATGAACAC | TGGTCCTTAGTGTCCTGGGT | NM_001004027.1 |
| *UCHL1* | TGCCTTTTCCGGTGAACCAT | AACGGGGATAAAGCGAAGGG | NM_213763.2 |
